# Eggplant Rootstock Grafting Enhances Tomato Fruit Sweetness and Nutritional Value via Metabolic Reprogramming

**DOI:** 10.1002/fsn3.71431

**Published:** 2026-01-14

**Authors:** Meiying Ruan, Xizhi Huang, Rongqing Wang, Yuan Cheng, Guozhi Zhou, Qingjing Ye, Zhuping Yao, Hongjian Wan, Zhimiao Li, Chenxu Liu, Chi Zhang

**Affiliations:** ^1^ State Key Laboratory for Quality and Safety of Agro‑Products Institute of Vegetables, Zhejiang Academy of Agricultural Sciences Hangzhou China; ^2^ Linping District Bureau of Agriculture and Rural Affairs of Hangzhou Municipality Hangzhou China; ^3^ State Key Laboratory for Quality and Safety of Agro‑Products, Institute of Virology and Biotechnology Zhejiang Academy of Agricultural Sciences Hangzhou China

**Keywords:** eggplant rootstock, fruit sweetness, grafting, metabolic reprogramming, tomato

## Abstract

Small tomatoes are an important economic fruit crop. Strategies to rapidly and stably improve their taste and nutritional quality are of significant economic value. This study evaluated grafting tomato scion ZheYingFen1 (ZYF1) onto eggplant rootstocks (ZheQie117 or ZheQie10). Comprehensive metabolomics and targeted assays demonstrated that grafting substantially increased fructose and glucose levels, thereby enhancing sweetness compared with self‐rooted controls. Lycopene content rose significantly, particularly with the ZQ117 rootstock, without elevating key organic acids linked to sourness. Conversely, most free amino acids decreased, including umami‐associated glutamate. Energy metabolite profiling showed graft‐specific shifts, with pronounced enrichment in sulfur‐containing glucosinolate biosynthesis pathways, suggesting modified defense responses and flavor profiles. These results demonstrate that compatible eggplant rootstocks provide a practical horticultural strategy to directly improve tomato sweetness and nutritional value without compromising acidity. This work provides evidence that eggplant rootstocks can effectively improve both the sweetness and nutritional value of tomatoes through metabolic reprogramming, offering a practical and sustainable approach for quality enhancement in commercial tomato production while maintaining a favorable acidity balance.

## Graphical abstract

Grafting tomato scion (ZYF1) onto eggplant rootstocks (ZQ117/ZQ10) reprograms fruit metabolism to enhance key quality traits. Metabolomic analyses revealed a significant increase in fructose and glucose levels, directly improving fruit sweetness, alongside a marked rise in the nutritional antioxidant lycopene. This quality enhancement was achieved without altering the fruit's organic acid content, maintaining a favorable acidity balance offering a sustainable strategy for commercial production.

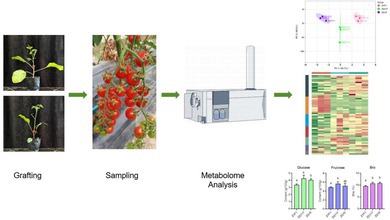

## 1 Introduction

Tomato is one of the most popular agricultural products with both vegetable and fruit attributes worldwide. With large‐scale industrial production, tomato varieties with improved external phenotypic traits such as size, color, and weight have been selected for long‐term storage and long‐distance transportation (Cai et al. 2024). As a trade‐off, desirable traits such as nutritional and taste quality may be compromised. Therefore, cultivating tomatoes with higher nutritional value or better taste can help meet changing market demands. The nutrition and taste of tomatoes are determined by various metabolites in the fruit, with key contributors including free amino acids (Chen and Zhang 2007), organic acids (Jia et al. 2022), polyphenols, and flavonoids (Xu et al. 2018). Thus, the content and ratio of different metabolites can be used to predict and measure the taste quality of tomato fruits.

Grafting is a widely used technique in horticulture and agriculture which can improve the disease resistance and quality of the scion. Tomato production is often limited by pathogens and abiotic stresses such as nutrient imbalances and drought, which seriously affect its yield and quality (Cui et al. 2021). Through grafting, scions are joined onto rootstocks that are adapted to the corresponding soil environment, which can effectively improve the adaptability of specific tomato varieties to local soil conditions. For example, interspecific grafting can enhance tomato resistance to gray leaf spot by altering epigenetic modifications (Liu et al. 2024). In terms of adapting to abiotic stress, self‐grafting can improve tomato water stress tolerance and phenolic content (Sanchez‐Rodriguez et al. 2012). Currently, most studies on tomato grafting focus on using different rootstocks to adapt to environmental stress, and research on nutrients is mostly confined to the determination of a few specific substances; more research is needed on the effect of grafting on the nutritional quality of tomato.

To effectively evaluate and enhance the nutritional and sensory qualities of agricultural products like tomato, metabolomics has emerged as a valuable analytical tool (Zhu et al. 2018). This approach enables comprehensive profiling of diverse metabolites in food matrices, offering insights into the complex biochemical composition that directly influences quality, environmental adaptability, and disease resistance (Karakas and Fernie 2025; Li et al. 2024; Singh et al. 2023). In food nutrition analysis, metabolomics facilitates the simultaneous quantification of key compounds—such as sugars, organic acids, amino acids, and phenolic compounds (Zhang et al. 2023). By mapping these metabolic signatures, researchers can objectively assess how agricultural practices, including grafting, alter the intrinsic quality attributes of crops beyond visual phenotypes. For tomatoes, metabolomics serves as a powerful tool, offering insights into how rootstock‐scion interactions alter metabolic pathways to improve dietary quality and consumer acceptance. In this study, we utilized metabolomics to reveal the effect of eggplant rootstock on reprogramming nutrient‐related metabolites of tomato fruits and found that grafting could enhance sugar and lycopene content without affecting the acidity of the fruits. This study provides a reference for a systematic understanding of the effects of grafting on tomato quality.

## 2 Materials and Methods

### 2.1 Plant Materials

The rootstock varieties are ZheQie10 (ZQ10) and ZheQie117 (ZQ117), while the scion is ZheYingFen1 (ZYF1). Tomatoes were grown in plastic single‐cell greenhouses in Haining City, China. The planting soil was slightly alkaline or neutral. Three replicates were set up, each with an area of 5 square meters, and 30 plants were planted in each replicate. Uniform fertilizer and water management were implemented. In each plot, 5 kg of decomposed sheep manure, 0.5 kg of compound fertilizer (N: P: K = 15:15:15), and 0.5 kg of calcium magnesium phosphate were applied.

### 2.2 Grafting

77‐day‐old rootstocks and 59‐day‐old scions that had not yet flowered were used for grafting. The grafting procedure was as follows: (1) Cutting the rootstock: A knife was used to make a horizontal cut above the second or third true leaf of the rootstock seedling. Then, a downward incision of 0.8–1.0 cm in length was made from the middle of the stem cross‐section. (2) Cutting the scion: A horizontal cut was made on the stem of the scion seedling. On both sides of the lower end of the scion stem, one cut each was made to form a wedge shape, approximately 0.3 cm thick. The cutting surface was 0.8–1.0 cm long and matched the size of the rootstock interface as closely as possible. (3) Connecting: The cut interface of the scion seedling was aligned with that of the rootstock seedling at the cambium layer, inserted into the rootstock cut, and ensured full contact between scion and rootstock. (4) Fixing the interface: After alignment, a square grafting clamp was used to secure the interface. The grafted seedling was placed in a prepared small greenhouse, covered with plastic film, and shaded to allow the graft union to heal.

### 2.3 Gas Chromatography–Mass Spectrometry Analysis for Sugar Metabolome

A 20 mg aliquot of freeze‐dried and ground sample powder was weighed into a labeled centrifuge tube. A 500 μL extraction solvent mixture of methanol: isopropanol: water (3:3:2, v/v/v) was added to each sample, followed by vortex mixing for 3 min and ultra‐sonication in a 4°C water bath for 30 min. After centrifugation at 12,000 r/min for 3 min (4°C), 50 μL of supernatant was collected and mixed with 20 μL of internal standard solution (1000 μg/mL). This mixture was dried under nitrogen and lyophilized. Derivatization was performed by adding 100 μL of methoxyamine salt in pyridine (15 mg/mL) and incubating at 37°C for 2 h, followed by addition of 100 μL BSTFA and further incubation at 37°C for 30 min. The derivatized solution (50 μL) was diluted with n‐hexane to 1 mL, filtered through a 0.22 μm membrane, and stored in amber vials for GC–MS analysis.

An Agilent 8890‐5977B GC–MS (Agilent Technologies, Santa Clara, CA, USA) was used for sugar metabolome analysis. GC–MS analysis utilized a DB‐5MS column (30 m × 0.25 mm × 0.25 μm) with helium carrier gas at 1 mL/min. A 1 μL sample was injected in split mode (5:1) with the following temperature program: initial hold at 160°C for 1 min, ramp to 200°C at 6°C/min, then to 270°C at 10°C/min, and finally to 320°C at 20°C/min (hold 5.5 min). The transfer line, ion source, and quadrupole temperatures were set at 280°C, 230°C, and 150°C, respectively. Electron impact ionization at 70 eV was employed with selected ion monitoring mode and a 4 min solvent delay. The extraction, detection, and quantitative analysis of metabolites were performed by Wuhan Metware Biotechnology Co. Ltd. (www.metware.cn).

### 2.4 Liquid Chromatography–Tandem Mass Spectrometry Analysis for Amino Acid and Energy Metabolism Related Metabolome

Approximately 50 ± 2.5 mg of each sample was weighed into a 2 mL centrifuge tube, with the exact mass recorded. Immediately after weighing, 500 μL of pre‐chilled (−20°C) 70% methanol–water extraction solvent and 400 μL of chloroform were added, followed by vortex mixing for 3 min. The mixture was centrifuged at 12,000 r/min for 10 min at 4°C, and 250 μL of the supernatant was transferred to a 1.5 mL centrifuge tube. The supernatant was stored at −20°C for 30 min, followed by recentrifugation under the same conditions (4°C, 12,000 r/min, 10 min). Finally, 180 μL of the resulting supernatant was aliquoted into an injection vial and stored at −20°C for subsequent analysis.

For amino acid analysis, an ExionLC AD UPLC coupled with a QTRAP 6500+ mass spectrometer (AB Sciex, Darmstadt, Germany) was employed. Chromatographic separation used an ACQUITY BEH Amide column (1.7 μm, 100 × 2.1 mm) with mobile phase A (water containing 2 mM ammonium acetate and 0.04% formic acid) and phase B (acetonitrile with 2 mM ammonium acetate and 0.04% formic acid). The gradient program was: 0–1.2 min (10% A), 9 min (40% A), 10–11 min (60% A), and 11.01–15 min (10% A) at a flow rate of 0.4 mL/min, column temperature of 40°C, and injection volume of 2 μL.

Energy metabolism analysis utilized a Waters ACQUITY H‐Class UPLC with the same QTRAP 6500+ MS (AB Sciex, Darmstadt, Germany). Separation occurred on an ACQUITY UPLC BEH Amide column (identical dimensions) with mobile phase A (water containing 10 mM ammonium acetate and 0.3% ammonia) and phase B (90% acetonitrile/water). The gradient was: 0–1.2 min (5% A), 8 min (30% A), 9.0–11 min (50% A), and 11.1–15 min (5% A), maintaining a flow rate of 0.4 mL/min, 40°C, and 2 μL injection volume.

Both methods shared identical MS conditions: ESI source at 550°C, positive/negative ionization voltages (±5500 V/−4500 V), curtain gas at 35 psi, and ion pairs detected via optimized declustering potential (DP) and collision energy (CE). The extraction, detection, and quantitative analysis of metabolites were performed by Wuhan Metware Biotechnology Co. Ltd. (www.metware.cn).

### 2.5 Determination of Sugar Content

The contents of fructose and glucose in tomato were determined using high‐performance liquid chromatography (HPLC) with refractive index or evaporative light scattering detection (ELSD), according to the Chinese National Standard GB 5009.8‐2023 (Method I). Briefly, homogenized samples were extracted with water. Precipitating reagents (zinc acetate and potassium ferrocyanide) were added, followed by ultra‐sonication and dilution. The extracts were filtered (0.45 μm membrane) after clarification. Separation was achieved on an amino‐bonded silica column (250 mm × 4.6 mm, 5 μm) using an isocratic mobile phase of acetonitrile: water (70:30, v/v) at 40°C. Quantification was performed using external calibration curves of standard sugars.

Sugar content (Brix %) was measured using a digital refractometer (PAL‐1, ATAGO Co., Japan). Prior to analysis, the prism was cleaned with distilled water and calibrated to zero. Approximately 0.3 mL of tomato juice was applied to the prism surface, ensuring full coverage without bubbles. Measurements were initiated via the “START” button, with results (Brix ±0.2%) and temperature recorded automatically after 3 s. Each sample was analyzed in triplicate, with prism cleaning repeated between replicates using distilled water.

### 2.6 Determination of Lycopene Content

Lycopene content was determined following the Chinese Agricultural Industry Standard (NY/T 1651‐2008). Lycopene was extracted from homogenized tomato samples using acetone–petroleum ether (1:1, v/v) under vacuum filtration. After liquid–liquid partitioning (for fresh/juice samples) or direct concentration (for paste), extracts were dried under nitrogen and reconstituted in dichloromethane. Analysis was performed by HPLC (C18 column, 250 × 4.6 mm, 5 μm) with UV detection at 472 nm. The mobile phase consisted of methanol–acetonitrile–dichloromethane (20:75:5, v/v) at 1.0 mL/min. Quantification used external calibration with lycopene standards (5–20 mg/L, purity ≥ 95%). Method sensitivity: LOD 0.13 mg/kg. Samples were analyzed in triplicate with blank controls.

### 2.7 Determination of Organic Acid Content

Total organic acids in tomato samples, expressed as malic acid or citric acid equivalents, were determined according to the Chinese National Standard GB 12456‐2021 (Method II). Homogenized tomatoes (25 g) were boiled for 30 min with 50 mL CO_2_‐free water, cooled, diluted to 250 mL with CO_2_‐free water, and mixed. If needed, the solution was filtered. A 25 mL aliquot was titrated using a calibrated pH meter (endpoint pH 8.2) with 0.1 mol/L NaOH standard solution under magnetic stirring. Blank titration was performed with CO_2_‐free water. Total acid content was calculated using the formula: Total acid content (g/kg) = (*c* × (*V*_1_ − *V*_2_) × *K* × 1000)/(*m* × *V*_a_), where *c* is NaOH concentration (mol/L), *V*_1_ and *V*_2_ are sample and blank titrant volumes (mL), *K* is the acid‐specific conversion factor (malic acid: 0.067; citric acid: 0.064), *m* is sample mass (g), and *V*_a_ is aliquot volume (mL). Triplicate analyses were conducted.

## 3 Result

### 3.1 Grafting Increases the Sweetness of Tomato

**FIGURE 1:**
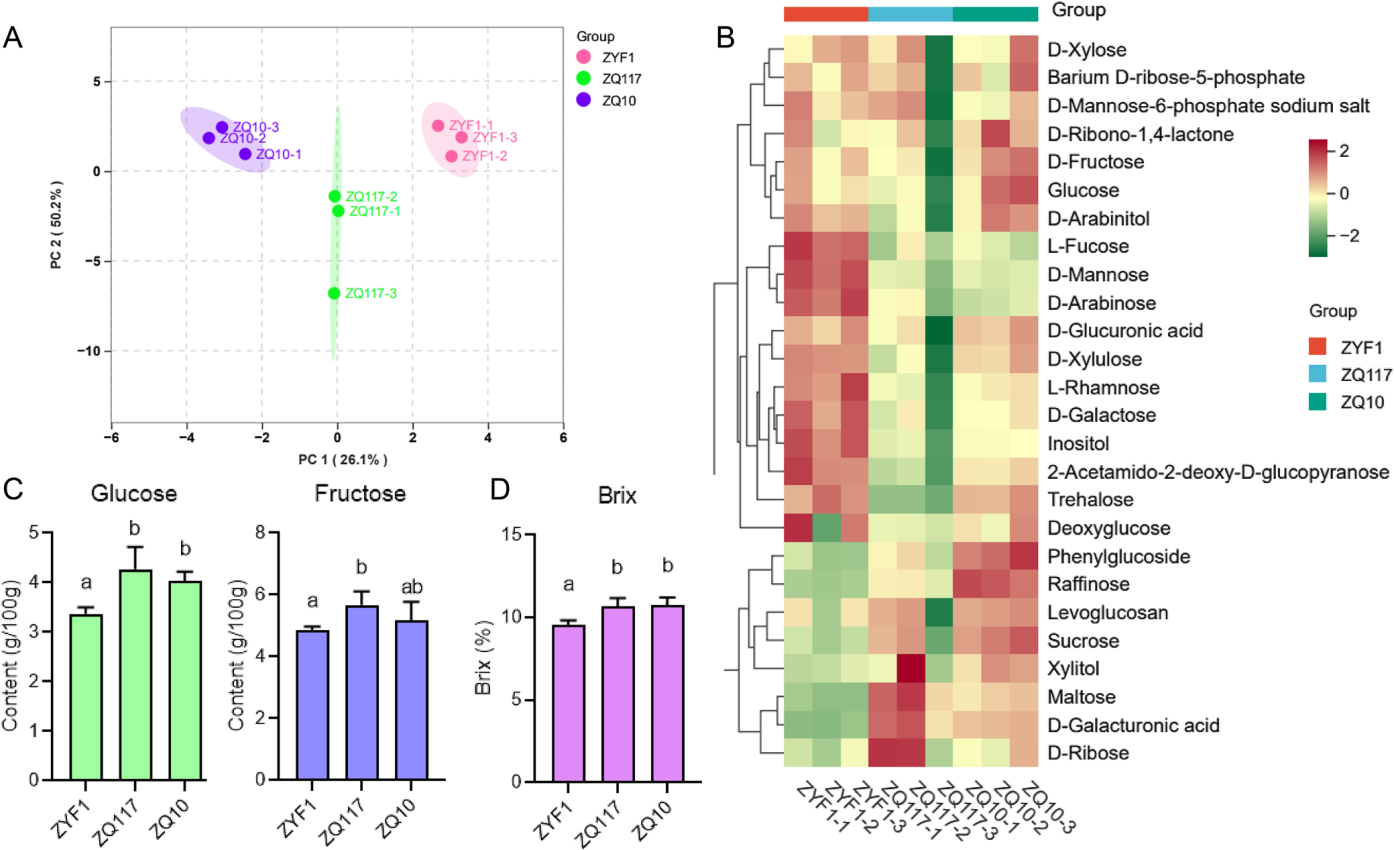
Sugar metabolomic analysis and sugar content. (A) Principal component analysis (PCA) of sugar metabolome. (B) Cluster heatmap of sugar in tomato fruits. (C) Glucose and fructose content in tomato fruit. (D) Sugar content of tomato fruit. Statistical significances were assessed by one‐way ANOVA (*p* < 0.05). Error bars indicate SD of three technical repeats. Different letters represent statistical differences for different samples.

To test the effect of grafting on tomato quality, two eggplant varieties, ZQ117 and ZQ10, were selected as rootstocks, and tomato ZYF1 was used as the scion. Grafting was performed when the rootstock seedlings were 77 days old and the scion seedlings were 59 days old. After transplanting into the greenhouse, when the third fruit cluster began to mature, the first fully ripe fruit from the second fruiting cluster was selected for metabolomic analysis (Table S1). To assess differences between groups and the consistency of biological replicates within the same group, principal component analysis (PCA) was conducted on the carbohydrate metabolome. The PCA data showed significant differences in sugar metabolism profiles between fruits from self‐rooted ZYF1 seedlings and the two grafted groups. The contribution rates of PC1 and PC2 were 26.1% and 50.2%, respectively (Figure 1A). A cluster heatmap clearly displayed the relative content of different sugars in the fruit. It was observed that the contents of disaccharides such as sucrose, maltose, phenylglucoside, and the trisaccharide raffinose were higher in grafted fruits. Most monosaccharides were more abundant in fruits from self‐rooted ZYF1 seedlings (Figure 1B). Notably, based on individual tests, the monosaccharides with the highest content and the greatest impact on sweetness—glucose and fructose—were present in higher amounts in grafted fruits compared to the self‐rooted control (Figure 1C). Additionally, the total sugar content of the fruits was measured using a refractometer. The results showed that the sugar content of the two grafted tomato fruits was significantly higher than that of the control group (Figure 1D). These results indicate that grafting mainly enhances the sweetness of tomato fruits by increasing the levels of soluble sugars, and provides a direct metabolic basis for the observed improvement in sensory quality.

### 3.2 Grafting Reduces Amino Acid Content

**FIGURE 2:**
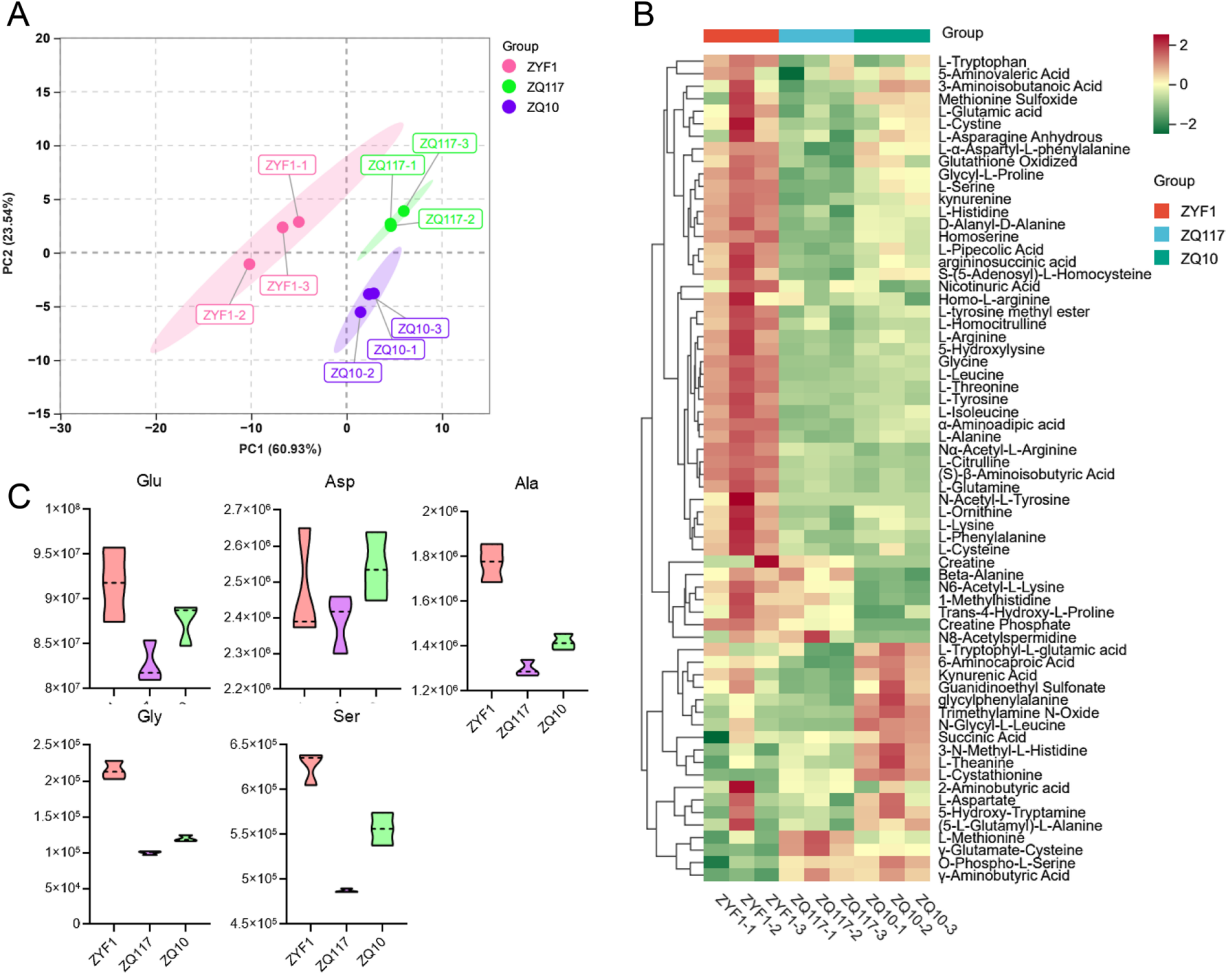
Amino acid metabolomic analysis. (A) Principal component analysis (PCA) of amino acid metabolome. (B) Cluster heatmap of amino acids in tomato fruits. (C) The relative content of several amino acids in the cluster heatmap.

Besides carbohydrates, amino acids also influence the taste of tomato fruits. Metabolomics analysis provided data on the impact of grafting on amino acid content (Table S2). PCA revealed significant differences in amino acid profiles between grafted and control tomatoes. The contribution rates of PC1 and PC2 were 60.93% and 23.54%, respectively (Figure 2A). A cluster heatmap was used to visualize the relative contents of amino acids and their derivatives in tomatoes from different groups. The results showed that the relative content of most amino acids was higher in the control group ZYF1 than in the two grafted tomato groups (Figure 2B). Among all amino acids, glutamate, which contributes to umami flavor (Albarracin et al. 2016), had the highest content. Other amino acids such as glycine and alanine also influence sweetness or umami (Bachmanov et al. 2016). Metabolomics data indicated that the contents of these amino acids were highest in the control group and were reduced by grafting (Figure 2C). In conclusion, the grafting plants resulted in a significant decrease in free amino acid levels, which may influence the umami taste of the tomato fruits compared to the self‐rooted plants. This reduction in amino acids occurred with the increase in sugars, indicating a metabolic reprogramming that rebalances the sweetness and umami.

### 3.3 Grafting Affects Energy Metabolism‐Related Metabolites

**FIGURE 3:**
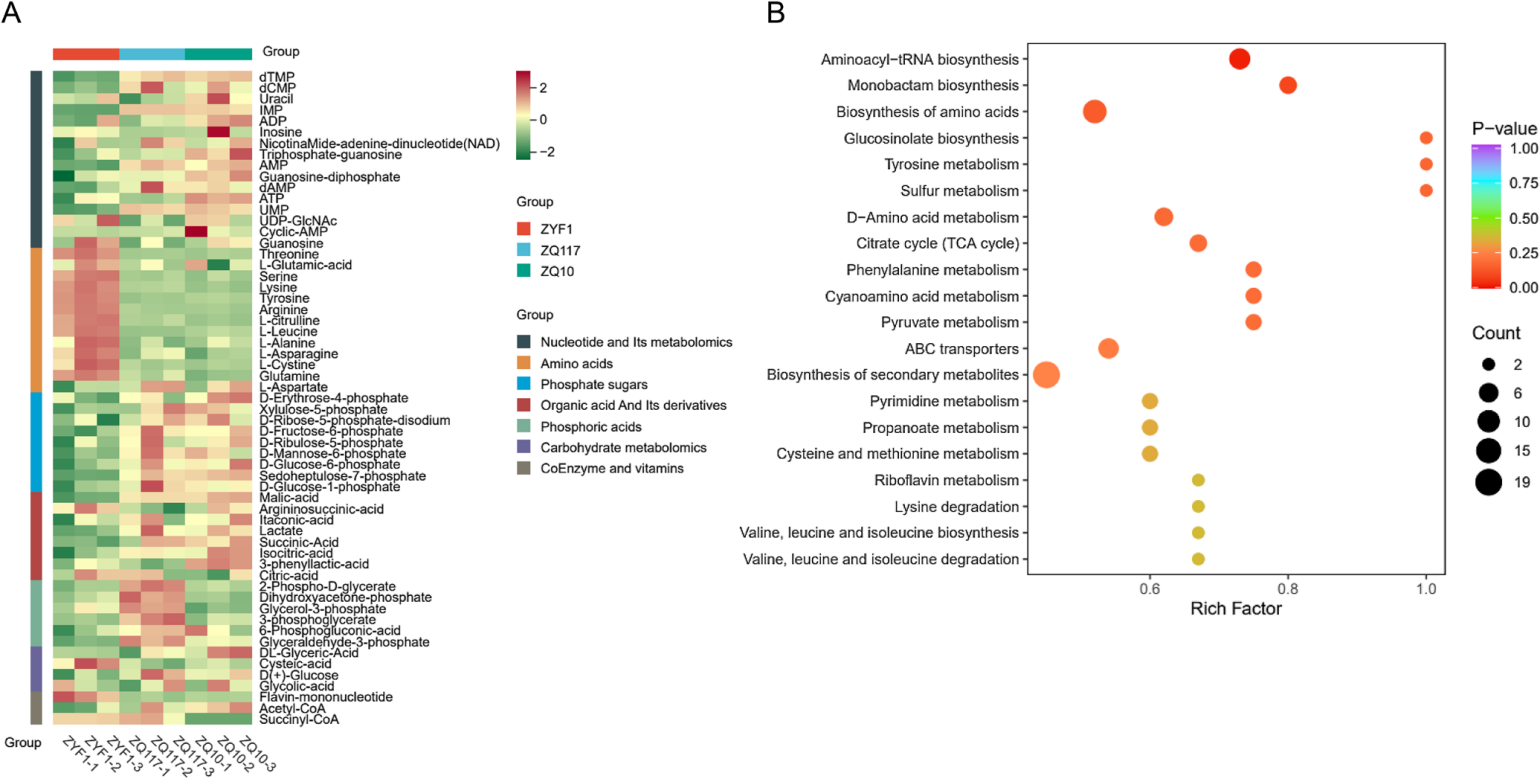
Metabolomic analysis of energy metabolites. (A) Cluster heatmap of energy metabolites in tomato fruits. (B) KEGG analysis of energy metabolites.

The growth and development of tomato fruits depend on energy metabolism. To explore the comprehensive effects of grafting on tomato metabolites, we analyzed metabolic profiles of various energy metabolism‐related substances (Table S3). These metabolites were classified into seven categories: nucleotides and their metabolites, amino acids, phosphate sugars, organic acids and their derivatives, phosphoric acids, carbohydrate metabolomics, and coenzymes and vitamins. Consistent with previous amino acid metabolism data, the control group ZYF1 had the highest amino acid content in the energy metabolism data. In the nucleotide category, the group grafted onto ZQ10 rootstock showed higher content. Both grafted groups had higher contents of phosphate sugars and organic acids and their derivatives. Among phosphoric acids, only the group with ZQ117 as rootstock had high content (Figure 3A). To further understand how grafting affects tomato metabolism, KEGG analysis was performed on significantly changed metabolites. The Rich factor represents the ratio of the number of differential metabolites in a pathway to the total number of metabolites annotated in that pathway. A higher value indicates greater enrichment. The three most enriched metabolic pathways were glucosinolate biosynthesis, tyrosine metabolism, and sulfur metabolism, while the pathway involving the biosynthesis of secondary metabolites had the largest number of enriched signal pathways (Figure 3B). Interestingly, glucosinolates are typically involved in plant responses to the environment, such as resistance to herbivores, and also confer unique flavors to plants (Mitreiter and Gigolashvili 2021). Glucosinolates are sulfur‐rich metabolites (Falk et al. 2007). Grafting‐induced changes in these resistance‐related metabolites not only alter the plant's environmental responses but also modify fruit taste.

### 3.4 Grafting Improves Lycopene and Organic Acids in Tomatoes

**FIGURE 4:**
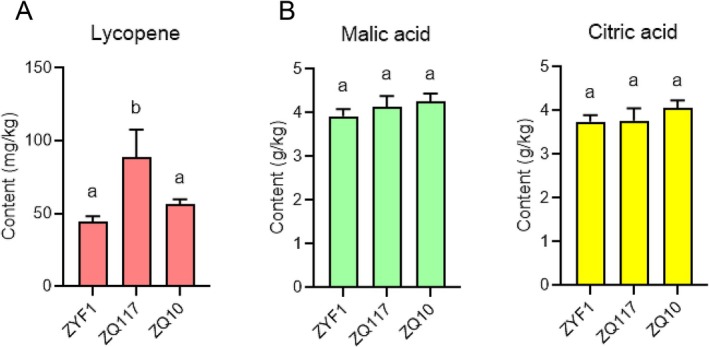
Content of lycopene and organic acid. (A) Content of lycopene. (B) Content of malic acid and citric acid. Statistical significances were assessed by one‐way ANOVA (*p* < 0.05). Error bars indicate SD of three technical repeats. Different letters represent statistical differences for different samples.

Lycopene is the most prominent lipophilic antioxidant in tomatoes, affecting both fruit color and human health. We detected lycopene content in tomatoes from different groups using HPLC. The results showed that lycopene content was higher in grafted tomatoes than in the control group ZYF1. Specifically, the group grafted onto ZQ117 rootstock showed a significant increase in lycopene content compared to the control (Figure 4A). Organic acids influence the sourness of tomato fruits, with malic acid and citric acid accounting for over 90% of organic acids in tomatoes (Quinet et al. 2019). The contents of malic acid and citric acid in different groups were determined by pH meter potentiometric titration. Consistent with previous metabolic data, both grafted groups had slightly higher contents of malic acid and citric acid than the control group, but the differences were not statistically significant (Figure 4B). These results indicate that grafting not only enhances the sweetness of tomato fruits but also significantly increases the content of the antioxidant compound lycopene without markedly increasing acidity. In conclusion, our findings demonstrate that eggplant rootstock grafting serves as a practical horticultural strategy to enhance tomato fruit quality by reprogramming key metabolites, leading to significantly increased sweetness and nutritional value via elevated fructose, glucose, and lycopene, without compromising acidity. This approach offers a sustainable alternative to conventional breeding for quality improvement in commercial tomato production.

## 4 Discussion

The findings presented here clearly demonstrate that grafting tomato (ZYF1) onto specific eggplant rootstocks (ZQ117 and ZQ10) significantly enhances fruit quality, primarily by elevating sugar content (including glucose and fructose) and the health‐promoting compound lycopene. This translates directly to a sweeter taste and increased nutritional value without substantially altering fruit acidity. This result differs from those of previous studies, indicating that the choice of rootstock significantly impacts tomato quality (Gong et al. 2022). While grafting is well‐established for improving disease resistance and abiotic stress tolerance in tomato (Fuentes‐Merlos et al. 2023; Spano et al. 2020), its impact on fruit metabolic composition and sensory quality requires deeper mechanistic understanding. In a multi‐omics study on the influence of rootstocks on tomato scions, it was found that proteins and metabolites from tobacco rootstocks would transfer to the scions, causing changes in genes related to biological rhythms and stress responses, as well as some metabolites (Ogawa et al. 2023). This provides clues for explaining how rootstocks alter the nutritional components of fruits.

The observed metabolic shifts raise crucial questions about the underlying mechanisms. How do eggplant rootstocks exert such influence on tomato fruit metabolism? The significant enrichment of sulfur‐containing glucosinolate biosynthesis pathways suggests a potential role for rootstock‐derived sulfur metabolites or signaling molecules transported via the graft union. Beyond specific metabolites, the transfer of small RNAs (sRNAs) from rootstock to scion represents another compelling avenue for future exploration (Goldschmidt 2014). These sRNAs could act as mobile signals, modulating gene expression and metabolic pathways in the tomato fruit, thereby altering sugar accumulation, amino acid profiles, and secondary metabolite synthesis like lycopene (Qing et al. 2022). Therefore, future research could focus on the sRNAs and metabolites in the phloem to investigate which substances from the rootstock influence the metabolic changes in the fruit. Phloem samples can be collected from both the graft union and the region near the fruit, followed by sRNA and metabolomic analyses. By comparing these with samples from non‐grafted tomato plants, it would be possible to identify rootstock‐specific metabolites or sRNAs that may contribute to reprogramming in the fruit metabolome.

Furthermore, while this study focused on quality parameters, the rootstock‐induced changes in glucosinolates—compounds strongly associated with plant defense responses—suggest that grafting may not only improve fruit quality but also potentially affect the plant's immunity (Mahmud et al. 2015). Therefore, evaluating the performance of these grafted combinations against common tomato pathogens and pests would be a valuable direction for future research. Similarly, given the documented role of grafting in enhancing drought tolerance (Zhang et al. 2019), it would be interesting for future research to specifically assess the resilience of the ZYF1/ZQ117 and ZYF1/ZQ10 combinations under abiotic stresses such as water deficit or elevated temperature. In summary, our study demonstrates that eggplant rootstocks can enhance tomato fruit sweetness by increasing sugar content and improve nutritional value by elevating lycopene levels. However, the underlying mechanisms and key factors responsible for this metabolic reprogramming require further investigation. Additionally, the effects of eggplant rootstocks on grafted plants' adaptability to biotic and abiotic stresses need to be clarified, as this understanding is crucial for evaluating the practical application potential of this grafting technique.

## 5 Conclusion

Utilizing compatible eggplant rootstocks offers a relatively simple and sustainable horticultural technique to directly improve the taste (sweetness) and nutritional content (lycopene) of commercial tomato varieties like ZYF1. This approach circumvents the need for lengthy breeding programs focused on complex quality traits. However, the observed reduction in amino acids, including key umami contributors like glutamate, warrants consideration. While the net effect on overall taste perception was a positive increase in sweetness, the specific impact on flavor needs consumer evaluation. Future research should include sensory panels and explore whether different rootstock–scion combinations or cultivation practices can optimize the balance between sweetness and umami. In conclusion, grafting onto eggplant rootstocks presents a promising strategy for enhancing tomato fruit quality. Future work must elucidate the root‐to‐fruit signaling mechanisms, explore the concomitant benefits for stress resistance, and assess consumer acceptance to fully harness this technique for producing higher‐value tomatoes.

## Author Contributions

**Meiying Ruan:** conceptualization, methodology, investigation, validation, resources, funding acquisition. **Xizhi Huang:** resources. **Rongqing Wang:** resources. **Yuan Cheng:** resources. **Guozhi Zhou:** resources. **Qingjing Ye:** resources. **Zhuping Yao:** resources. **Hongjian Wan:** resources. **Zhimiao Li:** resources. **Chenxu Liu:** resources. **Chi Zhang:** writing – review and editing, writing – original draft, investigation, conceptualization, methodology, data curation, formal analysis, visualization.

## Funding

This work was supported by Zhejiang Province Agricultural (Vegetable) New Varieties Breeding Major Science and Technology Project, 2021C02065.

## Supporting information


**Table S1:** fsn371431‐sup‐0001‐TableS1.xlsx.

**Table S2:** fsn371431‐sup‐0002‐TableS2.xlsx.

**Table S3:** fsn371431‐sup‐0003‐TableS3.xlsx.

## Data Availability

All data supporting the findings of this study are included in the manuscript and its Supporting Information.
